# Infants at risk for physical disability may be identified by measures of postural control in supine

**DOI:** 10.1038/s41390-021-01617-0

**Published:** 2021-06-26

**Authors:** Laura A. Prosser, Maria Ovando Aguirre, Susan Zhao, Daniel K. Bogen, Samuel R. Pierce, Kathleen A. Nilan, Huayan Zhang, Frances S. Shofer, Michelle J. Johnson

**Affiliations:** 1 Department of Pediatrics, University of Pennsylvania, Philadelphia, PA USA; 2 Division of Rehabilitation Medicine, The Children’s Hospital of Philadelphia, Philadelphia, PA USA; 3 Department of Bioengineering, University of Pennsylvania, Philadelphia, PA USA; 4 Department of Physical Therapy, The Children’s Hospital of Philadelphia, Philadelphia, PA USA; 5 Division of Neonatology, The Children’s Hospital of Philadelphia, Philadelphia, PA USA; 6 Department of Neonatology, Guangzhou Women’s and Children’s Medical Center, Guangzhou, Guangdong China; 7 Department of Emergency Medicine, University of Pennsylvania, Philadelphia, PA USA; 8 Department of Physical Medicine and Rehabilitation, University of Pennsylvania, Philadelphia, PA USA

## Abstract

**Background:**

Early detection of delay or impairment in motor function is important to guide clinical management and inform prognosis during a critical window for the development of motor control in children. The purpose of this study was to investigate the ability of biomechanical measures of early postural control to distinguish infants with future impairment in motor control from their typically developing peers.

**Methods:**

We recorded postural control from infants lying in supine in several conditions. We compared various center of pressure metrics between infants grouped by birth status (preterm and full term) and by future motor outcome (impaired motor control and typical motor control).

**Results:**

One of the seven postural control metrics—path length—was consistently different between groups for both group classifications and for the majority of conditions.

**Conclusions:**

Quantitative measures of early spontaneous infant movement may have promise to distinguish early in life between infants who are at risk for motor impairment or physical disability and those who will demonstrate typical motor control. Our observation that center of pressure path length may be a potential early marker of postural instability and motor control impairment needs further confirmation and further investigation to elucidate the responsible neuromotor mechanisms.

**Impact:**

- The key message of this article is that quantitative measures of infant postural control in supine may have promise to distinguish between infants who will demonstrate future motor impairment and those who will demonstrate typical motor control.
- One of seven postural control metrics—path length—was consistently different between groups.
- This metric may be an early marker of postural instability in infants at risk for physical disability.

## Introduction

Early detection of developmental delay or impairment in motor function is important to guide clinical management and inform prognosis during a critical window for the development of motor control^1^. The first 2 years of life are a critical period of neuroplasticity in the motor control centers of the brain^2^, and there is promising evidence that, when delivered early in life, motor training interventions are effective in reducing motor impairment^3^. This underscores the need for the early detection of impairment, and this need is widespread, with 5–10% of children suffering from developmental disabilities^4^. Cerebral palsy (CP), in particular, is the most common cause of lifelong physical disability in children, with an incidence of 2–3 per 1000^5–7^.

There is no widely adopted approach to the early detection of motor delay or impairment. Treatment for motor delay (later than typical achievement of motor skill milestones, such as sitting and walking) and motor impairment (dysfunction in the physical ability to move) are different^1^ but challenging to discriminate early in life. Discriminating these in the early months of life will inform clinical decision making at a critical time in development. Current clinical tests have poor predictive value^8^ or require extensive expertise, training, and effort to implement a battery of assessments and therefore are only accessible to infants in high-resource environments^9^. Consequently, there is a critical need to develop broadly accessible, quantitative methods to identify motor impairment in infancy that may predict future physical function.

**Fig. 1:**
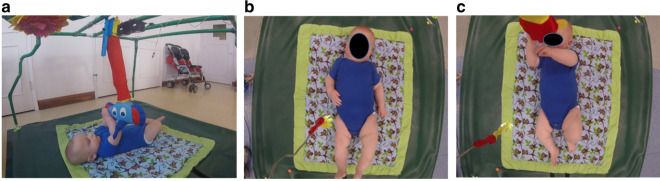
An infant lying supine in the toy gym during the recording of center of pressure (COP). **a** Side view of the infant playing with the hanging elephant toy; **b** aerial view of the infant in the no toy condition; **c** aerial view of the infant in the orangutan toy condition, designed to encourage bilateral reaching.

We have developed and pilot tested an instrumented infant play gym to quantify movement characteristics during natural play in the first year of life^10,11^. We now call the gym PANDA—for Play And NeuroDevelopmental Assessment—and are evaluating the potential utility of different measures from PANDA in developmental screening and early detection of infant motor impairment. One aspect of PANDA is the recording of center of pressure (COP) from an instrumented mat while infants lay supine (Fig. 1). COP measures have been used to quantify postural control, a key aspect of motor function, in typically developed young children^12^, children with CP^13^, and infants born full term and preterm^14,15^. Previous studies have reported different patterns of COP between infants born preterm and full term^14,16^, but there is no evidence to date of the ability to distinguish between children with and without future motor impairment using measures derived from COP data.

The purpose of this study was to investigate the ability of biomechanical measures of early postural control to distinguish infants with future impairment in motor control from their typically developing peers. We also report the influence of different toy conditions on postural control in the participant sample.

## Methods

### PANDA gym design

The PANDA gym includes an array of toys with sensors to measure play interaction, a camera-based computer vision system to measure limb and trunk kinematics, and a mat structure to measure the postural control of the infant (Fig. 1). The mat platform is a 4 ft × 4 ft carbon fiber foam core dragon plate (DragonPlate, Elbridge, NY) covered with foam padding for the infants’ comfort. A PVC structure is placed on top of the platform to suspend the toys and support the video system. The three sensorized toys are each designed to encourage a different type of infant motor interaction—unilateral arm reaching (elephant), bilateral arm reaching (orangutan), and leg kicking (lion). The toys provide visual and auditory feedback to encourage infant interaction.

### Instrumented mat specifications and validation

COP is determined from load cell (OMEGA Engineering, Model LC302–50; maximum load = 50 lbs) measurements taken at the four corners of the mat. The load cells are interfaced with signal conditioners with built-in 10 Hz low-pass filters (OMEGA Engineering, Model DRC-4710); and the four load signals, collectively measuring COP, are then sampled by an Arduino microcontroller at 60 Hz. These digitized measurements are transmitted by a serial connection to a PC computer, which stores the data onto the hard drive. Custom software was written in Python to perform this data transfer.

COP is calculated, with respect to two perpendicular axes, by taking the difference between the load cell readings along the axis and then normalizing with respect to the distance between the axis load cells and the weight of the infant. The calculation is performed through the combination of analog signal conditioner settings and digital computation in the microcontroller. The system is calibrated from test weights placed in different locations on the mat.

Precision and accuracy tests were conducted to assess the validity of the mat system. Precision tests measured the *X* and *Y* position of one marker relative to another by conducting four trials of placing calibrated weights in different locations and plotting the recorded data using a scaling procedure achieved through calibration tests. We determined the relative point-to-point distance precision to be within 2.2 cm. To determine how accurately a location physically measured on the mat translated to processed data, we placed weights at marked locations on the mat. Four trials of this test were conducted with weights of 5, 7, 9, and 11 kg. We analyzed the error bounds relative to weight. In both the *X* and *Y* directions, the amount of error and weight were inversely related (*r* = −0.86 and −0.95, respectively). Infants in our participant sample ranged in weight from 4 to 8 kg, and thus we infer an error range of about 3.5–5 cm.

### Participants

Infants born full term were recruited from The Children’s Hospital of Philadelphia (CHOP) and University of Pennsylvania (UPenn) communities and local childcare facilities. These infants were born at a gestational age of >37 weeks, were between the ages of 3–11 months at the time of testing, and had no history of significant cardiac, orthopedic, or neurological conditions. Infants who could walk were excluded. The majority of infants born pre-term had severe bronchopulmonary dysplasia (BPD) and were recruited from CHOP’s Newborn and Infant Chronic Lung Disease Program at the Harriet and Ronald Lassin Newborn/Infant Intensive Care Unit. Several infants born pre-term were also recruited from CHOP’s outpatient rehabilitation services. These infants were born at a gestational age of <36 weeks and were also between 3 and 11 months of age. Parents of eligible participants provided written informed consent. The human subject’s ethics committees at both UPenn and CHOP approved this study.

### Data collection

Timing of data collection was coordinated with caregivers, and when applicable, nursing staff, to avoid testing during regular meal or nap times. Prior to each data collection session, mat calibration was conducted by placing a calibration weight on each of the force sensors (one on each of the four corners of the mat) for 10 s. After calibration, a baseline condition was collected with no weight on the mat. Infant testing consisted of several trials for different toy conditions (no toy, elephant, orangutan, lion). Each trial was approximately 2 min in duration. The infant was laid in a supine position in the center of the mat at the beginning of each trial and removed between trials. The no toy condition was tested first (Fig. 1b), followed by randomized presentation of the toy conditions, determined prior to testing (Fig. 1c). Toy order was reversed for infants tested twice. For the elephant and orangutan trials, designed to encourage arm reaching, the toys were hung directly above the infant’s chest and adjusted vertically to hang easily within arm’s reach. The lion toy, designed to encourage leg kicking, was hung above the infant’s knees and adjusted vertically to hang within leg’s reach. Once a trial started, external stimuli were minimized. If the infant did not interact with the toy after 28 s, the toy would produce auditory stimulation to bring the infant’s attention to the toy. If present, the caregiver was able to sit nearby the infant (outside the boundaries of the mat). If the infant displayed hesitancy or became upset, the caregiver, a research physical therapist, or a nurse would talk to the infant to reassure him/her. If the infant could not be consoled while lying on the mat, the infant was picked up and the trial was stopped.

Video and postural data of each trial were collected along with toy sensor data. Videos were reviewed to determine acceptable trials (or acceptable segments of trials lasting at least 30 s). Acceptable trials were based on the following criteria: (1) infant was not being touched, (2) infant did not cry, (3) infant remained in a supine position within the bounds of the mat (i.e., did not roll, sit, or crawl), and (4) infant was awake and alert (Brazelton stage 4 or 5). Postural control data were included in the analysis if acceptable data were collected for the No Toy condition and at least one toy condition.

### Data processing

**Fig. 2:**
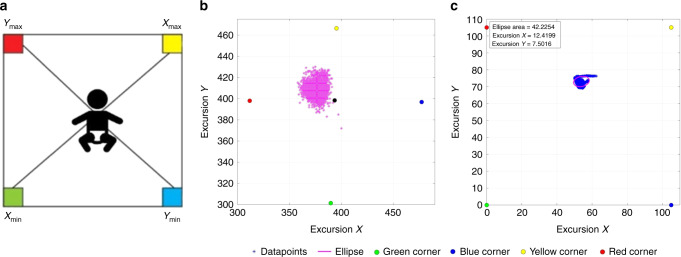
Postural control data processing. **a** Schematic of infant lying supine on the instrumented mat. **b** Example raw data. The Green load cell was wired to be the minimum *Y* value, and the Yellow load cell as the maximum *Y* value. The Red load cell relates to *X*-min., and the Blue as *X*-max. We hardcode these bounds to be 104 × 104 cm, the measured length along the edges from one load cell to the other. **c** Representative stabilogram with the raw data calibrated to real-world coordinates and filtered. The data were translated and rotated so the green corner was positioned at (0,0). Actual values for ellipse area and excursions in *X* and *Y* are listed.

Postural control data were processed using the Matlab software (Mathworks, Inc.). The baseline control data were first processed to determine the range of values from the force sensors that define the four corners of the mat. Infant test trial data were then compared against the baseline control trial to correct for offset, and signals were scaled by the weight ratio of the baby’s weight to the calibration weight, then in relation to the minimum and maximum values from the calibration trial and rotated such that *X* aligned with the infant’s medial–lateral axis and *Y* aligned with the infant’s caudal–cephalic axis. This process outputs the COP position of the infant with respect to real-world coordinates relative to the location of the four sensors and the bounds of the mat. Movement in the *X* direction represents side-to-side shifting and movement in the *Y* direction represents cephalocaudal (head-to-toe) vertical shifting. See Fig. 2 for an example of the postural control data processing steps.

After calibration to “real-world” coordinates, postural control data were smoothed using a 100-point moving average filter to remove noise from the recorded signal^17^. This moving-average filter has a nominal 3 db cutoff frequency of 2.7 Hz^18^, but with a gradual, rather than sharp, roll-off in frequency response^17^. This filtering technique was applied to the *X* and *Y* time series.

### Measures for analysis

*Postural control* variables were derived from the COP time series for each trial. We calculated seven postural control metrics (see Table 1) that are routinely reported in the literature to quantify postural control^19^. These postural control metrics are COP magnitude, root mean square (RMS) error from mean COP, excursion of COP and standard deviation of COP in *X* (COPx) and *Y* (COPy) directions, path length, and ellipse area approximation of the scatter of COPx versus COPy. Table 1 shows the postural control variables and their description.

**Table 1:** Postural control variables, derived from center of pressure time series data.

Measure	Operational definition	Unit of measure
RMS	The root mean square of the norm (Euclidean distance) of the COP vectors; the sum of deviations from the mean COP	cm
ExcursionX	The farthest distance (magnitude) in the real-world *X* direction, or medial–lateral	cm
ExcursionY	The farthest distance (magnitude) in the real-world *Y* direction, or caudal–cephalic	cm
StdDevX	Standard deviation of the COPx values	cm
StdDevY	Standard deviation of the COPy values	cm
PathLength	The total path length distance calculated, time normalized to 1 min	cm
EllipseArea	The area of an ellipse fit to the data, such that 95% of the data points are captured in the ellipse area	cm^2^

*Demographic* variables used for analysis were infant age (corrected for prematurity if applicable), birth status (full term or preterm), and outcome after age 2 years (Impaired or Typical motor control). Outcome after age 2 years was determined from medical record review by an experienced pediatric physical therapist for all infants born preterm (*n* = 10). For infants born full term, outcome was determined by verbal contact with parents when possible (*n* = 10) or assumed normal if future parental contact was not made (*n* = 9, infants tested in childcare facilities). Children who demonstrated delayed gross motor skills but did not have signs of abnormal motor control (such as spasticity or stereotypical movement patterns) were classified as typical motor control.

### Statistical analysis

To determine differences in the seven postural control metrics by birth status or motor control at 2 years of age, a two-factor analysis of variance in repeated measures was used where birth status or motor control outcome was the grouping factor and toy condition was the repeated measure. To minimize type I error, post hoc pairwise comparisons using Tukey–Kramer tests with adjusted *p* values were performed to examine differences between toy type and birth status or motor control. Ellipse area data were not normally distributed and were log transformed prior to analysis. All analyses were performed using the SAS statistical software (Version 9.4, SAS Institute, Cary, NC).

## Results

### Participant sample

Twenty-nine infants were tested, 19 were born full term and 10 were born preterm. Data from 11 of the infants born full term (and all over the age of 7 months) were excluded from analysis because the infants either rolled or crawled on the mat or they were fussy and were not able to be calmed while laying supine without being touched. Data from three of the infants born preterm were excluded because of technical issues with the mat on the day of testing. Our final dataset included 8 infants born full term (mean age 4.8 months) and 7 infants born preterm (mean corrected age 3.5 months). All 8 of the infants born full term and 3 of the infants born preterm demonstrated typical motor control at age ≥2 years. Four of the infants born preterm demonstrated impaired motor control at age ≥2 years (Table 2). There was no difference in age (when corrected for preterm birth, if applicable) or weight between the groups.

**Table 2:** Demographic characteristics of participant sample.

Group by birth status	Sex	Mean age in months (corrected for preterm birth in the Preterm group, range)	Mean weight in kg (range)	Outcome at 2 years
Full term (*n* = 8)	3 males, 5 females	4.8 (4.0–6.5)	6.7 (5.5–8.2)	Typical motor control (*n* = 8)
Preterm (*n* = 7)	5 males, 2 females	3.5 (1.0–5.5)	6.1 (4.1–8.6)	Typical motor control (*n* = 3), impaired motor control (*n* = 4)

### Postural control

#### Postural control by birth status

**Fig. 3:**
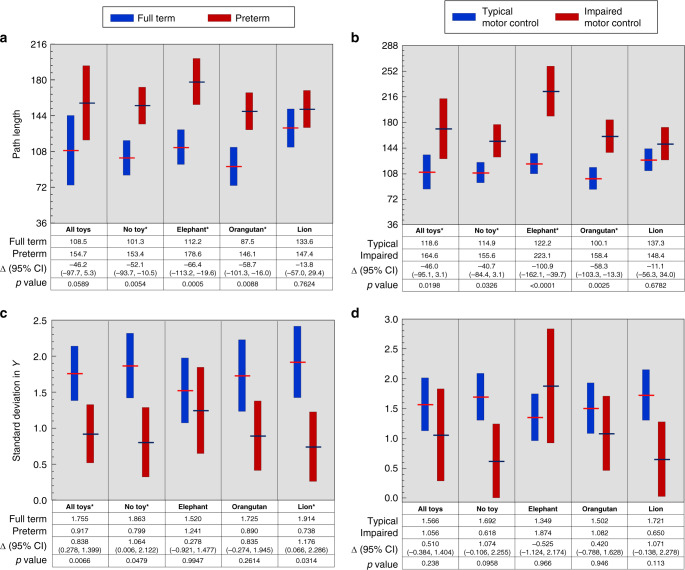
Group differences in postural control. Group data for Path Length (**a**, **b**) and Standard Deviation in Y (StdDevY; **c**, **d**) for all toy conditions combined and individually. Group comparisons for Full Term vs. Preterm birth are on the left. Group comparisons for future Typical vs. Impaired motor control are on the right. Group differences were considered significant if *p* < 0.05 and are indicated by asterisk (*) following the condition name. Path length distinguished between groups by birth status and by later motor control for most toy conditions, with the Preterm and Impaired motor control groups having higher path length than the Full Term and Typical motor control groups, respectively. StdDevY distinguished between groups by birth status in the no toy and lion (kicking toy) conditions, with the Preterm group having lower variability in COPy than the Full Term group. Similar differences were observed when grouped by later motor control, but these differences did not reach significance (perhaps due to low statistical power as a result of the small sample size in the Impaired motor control group).

Counter to our hypothesis, PathLength was significantly higher in the Preterm group compared to the Full Term group in 3 toy conditions (no toy 153.4 vs. 101.3 cm, *p* = 0.0054; orangutan—bilateral reach 146.1 vs. 87.5 cm, *p* = 0.0088; and elephant—unilateral reach 176.6 vs. 112.2 cm, *p* = 0.0005) but not for the lion—leg kick toy (147.4 vs. 133.6 cm, *p* = 0.7624), as shown in Fig. 3a. More consistent with our hypothesis, ExcursionY was significantly lower in the Preterm group (mean = 7.3 cm) compared to the Full Term group (mean = 11.0 cm) regardless of toy condition (difference = 3.65 cm, 95% confidence interval (CI): 0.13–7.17 cm, *p* = 0.043). Similarly, StdDevY was also significantly lower in the Preterm group (mean = 0.92 cm, *p* = 0.007) compared to the Full Term group (mean = 1.76 cm) in all toy conditions combined and in the no toy (0.80 vs. 1.86 cm, *p* = 0.048) and lion (leg kick) toy (1.18 vs. 1.91 cm, *p* = 0.031) conditions but not in the elephant or orangutan (arm reaching) toy conditions (Fig. 3c). Additionally, EllipseArea was significantly lower in the Preterm vs. Full Term group (difference = 2.3 cm, 95% CI: 1.06–4.84 cm, *p* = 0.038). However, these differences were small (1.06–2.41 cm) for most toy conditions, except the orangutan—bilateral reach (mean 4.7 cm, 95% CI: 1.07–20.9 cm). There were no differences between the Full Term and Preterm groups for any toy condition for RMS, ExcursionX, and StdDevX. In the no toy condition, of the seven measures, only two demonstrated differences between groups. PathLength was significantly higher and StdDevY was significantly lower in the Preterm vs. Full Term group (Table 3).

**Table 3:** Group comparisons for postural control variables in the no toy condition.

	Birth status			Motor control outcome		
	Full term (*n* = 8)	Preterm (*n* = 7)	*p* value	Typical (*n* = 11)	Impaired (*n* = 4)	*p* value
RMS (cm)	7.033 (3.527)	3.598 (1.550)	0.346	6.429 (3.377)	3.540 (2.060)	0.703
ExcursionX (cm)	10.600 (5.687)	10.643 (4.178)	>0.999	10.577 (5.098)	9.085 (3.858)	>0.999
ExcursionY (cm)	10.690 (3.396)	5.960 (3.412)	0.436	9.615 (3.894)	4.512 (0.555)	0.353
StdDevX (cm)	1.510 (0.872)	1.193 (0.698)	0.999	1.393 (0.815)	1.207 (0.902)	>0.999
StdDevY (cm)	1.863 (0.920)	0.799 (0.502)	0.048*	1.692 (0.944)	0.618 (0.303)	0.096
PathLength per minute (cm)	101.3 (40.4)	153.4 (53.3)	0.005*	114.9 (55.5)	155.6 (15.4)	0.033*
EllipseArea (log10)	3.117 (1.230)	2.615 (1.258)	0.743	2.994 (1.229)	2.395 (1.234)	0.610

Values are groups means (SDs). Ellipse area data were log transformed for analysis to accommodate for skewness. *p* Values were adjusted for multiple comparisons using Tukey-Kramer tests and statistical difference between groups is indicated by asterisk (*) (*p* < 0.05).

#### Postural control by outcome after 2 years of age

We observed similar patterns of postural control when participants were grouped by outcome after 2 years of age as to when they were grouped by birth status. Consistent with the results by birth status, PathLength was higher in the group with later Impaired Motor Control compared to the group with later Typical motor control in all toy conditions (no toy 155.6 vs. 115.9 cm, *p* = 0.033; orangutan—bilateral reach 158.4 vs. 100.1 cm, *p* = 0.003, and elephant—unilateral reach 223.1 vs. 122.2 cm, *p* < 0.0001) except for the lion—leg kick toy (148.4 vs. 137.3 cm, *p* = 0.678), as shown in Fig. 3b. There were no differences between groups with Typical and Impaired motor control after 2 years of age for any toy condition for RMS, EllipseArea, ExcursionX, StdDevX, ExcursionY, or StdDevY. However, group differences in StdDevY followed a similar pattern as to when participants were grouped by birth status, with the later Impaired motor control group demonstrating lower variability in COPy than the Typical motor control group in the no toy (0.62 vs. 1.70 cm, *p* = 0.096) and lion—leg kick toy (0.65 vs. 1.72 cm, *p* = 0.113) conditions but not to a level that reached statistical significance between the motor control groups (see Fig. 3d). In the no toy condition, only PathLength was significantly different between groups and was higher in the Preterm vs. Full Term group (Table 3).

## Discussion

Infants with significant early postnatal morbidities, such as preterm infants with severe BPD, are at great risk of developmental delay. Early identification and intervention of motor delay in these infants has been difficult. This study demonstrates that quantitative methods of measuring postural control in infants born preterm and who are still hospitalized are feasible and show promise for the early detection of motor impairment. Postural control, specifically COP path length, is a measure that may have potential to discriminate between infants who are at risk of motor impairment or physical disability and infants who will demonstrate more typical development of motor control.

While the result of higher path length in the group with later impaired motor control compared to the group with later typical motor control was counter to our hypothesis, there are some suggestions in previous literature that lower path length may be representative of greater postural control. Fallang et al. reported a reduction in COP path length, or “total COP displacement,” with increasing supine postural control in typically developing infants at 6 months of age compared to at 4 months of age^20^. The displacement occurred primarily in the medial–lateral direction compared to the cephalo-caudal direction. Dinkel et al. reported path length in sitting as “Sway Path” in a group of 14 healthy, normal weight infants^21^. Postural control COP data were collected at the onset of independent sitting skill and 1 month after. While the authors did not report a statistical comparison between time points, they report an 8% reduction in sway path from sitting onset to 1 month later, a period time over which postural control improves in typically developing children. Also of note are the observations by Donker et al. that 10 children aged 5–11 years with CP demonstrated greater path length during static standing than their typically developing peers^22^.

Perhaps most relevant is the work of Støen and colleagues who analyzed supine infant movement from video recordings and interpreted their findings relative to an observational clinical classification of high risk for CP (termed “absent fidgety” movements)^23^. One measure was the standard deviation of the movement of the centroid over the duration of the recording. The centroid is the spatial center of the moving pixels, the center of the infant’s movements, and high variability (standard deviation) of this centroid motion represents the degree of unsteadiness during movement, independent of movement amplitude (excursion). Infants with absent fidgety movements demonstrated greater variability in the centroid movement. While this approach used a slightly different calculation than our COP path length measure, both findings may be reflective of unsteadiness or instability in movement behavior.

These reports from existing literature suggest that a reduction in path length represents improved postural control (i.e., greater postural stability). In the current study, we observed consistently higher path length values in infants at risk for motor impairment, whether grouped by birth status or future motor control, for all toy conditions except the lion toy (designed to encourage kicking rather than reaching and placed above the infants’ knees rather than above the chest). Therefore, path length may be an early marker of motor impairment and specifically an early marker of impaired postural control that can be measured longitudinally over development and in different postures. Our observations also suggest that the addition of a suspended toy to encourage reaching or kicking may not be necessary to identify differences in this measure. However, the presence of a toy does not guarantee that the infant interacted with the toy, and therefore analyzing COP in the context of specific play behaviors could offer additional insight.

No other postural control measure demonstrated a consistent potential to identify early motor impairment. ExcursionY, StdDevY, and EllipseArea were lower in the Preterm group compared to the Full Term group for select toy conditions, and EllipseArea was lower in the group with later Impaired motor control compared to the group with later Typical motor control for all toy conditions combined but no single toy condition alone. While these results were closer to our hypothesis that infants with greater postural control would demonstrate larger values on all COP measures, the group differences for these measures were not consistent enough to likely be useful in predicting future development of motor control. However, given the low statistical power from our small sample, the trends in StdDevY and EllipseArea should continue to be investigated.

One reason for observing more group differences in the cephalo-caudal (*Y*) direction measures than the medial–lateral (*X*) direction measures may be that we excluded trials when infants rolled to one side, which would impact excursion and variability (StdDev) in the *X* direction a great deal. Arm reaching would also impact COP, primarily in the medial–lateral (*X*) direction but to a lesser degree than rolling. In contrast, leg kicking was not excluded and would be the primary behavioral pattern to greatly impact excursion and variability (StdDev) in the *Y* direction. We suspect that leg kicking behavior was the reason for observing a group difference by birth status in StdDevY during the lion toy condition, which was specifically designed and positioned to encourage leg kicking. With a larger sample, it is possible that a group difference by later motor control outcome would have been observed as well.

Our results for the RMS measure are generally consistent with Dusing et al.’s findings across three previous studies. When comparing postural control between younger infants born preterm or full term^14^, there was no difference between groups in the RMS resultant in infants at 1–3 weeks of age (corrected for preterm birth as applicable). When analyzing RMS in the *X* and *Y* directions separately (which we did not replicate), the authors observed larger RMS values in the Preterm group compared to the Full Term group in just the caudal–cephalic (*Y*) direction. In the two groups of older infants, with a similar age range as the current study, this same research group observed no group difference in RMS in infants born preterm compared to full term during the development of head control and just a small group difference during the development of reaching skill^16^. In the cohort of 22 older infants who were born full term, the researchers observed that RMS was lower in a toy condition compared to a no toy condition in both caudal–cephalic (*Y*) and medial–lateral (*X*) directions^24^. This last result is different from our observations that found no effect of toy condition on RMS resultant but may be explained by our smaller sample size. In general, we agree with Dusing and colleagues that the RMS measure, a measure of the magnitude of variability in the postural control time series, is of limited usefulness in the early detection of motor impairment^16^.

Taken together, it is at first unexpected to observe consistently higher path length in the infants at risk for motor impairment without corresponding higher values for other measures of COP magnitude such as excursion, variability (StdDev), and ellipse area. However, this perhaps may be part of the discriminate potential of quantifying early postural control. A large path length appears to represent postural instability, especially when movement cannot be controlled enough to generate large excursions in COP (through weight shifts side to side or lifting the extremities against gravity off the surface). Infants at risk for impaired motor control may demonstrate early signs of motor impairment through repeated, small-amplitude, unsteady movements within a small circumference. In contrast, infants with typical development of motor control may be able to generate a smoother path while controlling movement over a larger excursion. Consider an adult with poor standing balance who is unsteady while standing in place, compared to an active adult who is able to maintain dynamic standing balance (reaching, squatting, rotating the trunk) with ease. The former is unsteady but relatively still, while the latter is controlled over a wide range of movement. In fact, robust postural control is not simply the ability to maintain a static posture, but the ability to control or adapt postural control during a variety of movements and environmental contexts. A progressive increase in the smoothness of movement has been reported in the development of reaching skill^25,26^, and less smoothness during movement (which has sometimes been referred to as “jitteriness”) has been observed during crawling in infants with developmental delay compared to those with typical development^27^. Therefore, it may be of particular note that the direction of differences between groups in our study was opposite between PathLength (perhaps a measure of stability) and the excursion and variability measures (Excursion, EllipseArea, StdDev—perhaps measures of dynamic range). This opposite direction pattern may have discriminatory or predictive potential when the variables are used in combination and should be further explored.

This analysis includes a smaller sample than our full dataset as a result of excluding trials with infants who were fussy or did not remain in supine or as a result of technical issues. Excluding the infants with greater mobility who did not remain in supine likely biases our group comparisons to be more similar to each other. Thus, any differences observed are more likely to be representative of motor control than simply movement behavior during testing. Additionally, younger infants with less mobility, prior to when motor developmental milestones are missed (such as sitting), are the target of early detection efforts, and therefore the loss of data from older more mobile children is fairly inconsequential. On the other hand, the neonatal intensive care unit may have introduced environmental factors that contributed to behavioral differences in movement, such as movement discouragement from monitoring leads, and supplemental feeding and breathing equipment. The resulting group of children with future impaired motor control was small (*n* = 4), which limited statistical power. It was valuable to have these 2-year follow-up outcome data for our sample, and it is possible that group differences in more postural control measures or in more toy conditions would be identified with a larger sample. An additional limitation is that our groups were not perfectly matched by age. The Preterm and Impaired motor control groups were approximately 1 month younger than the Full Term and Typical motor control groups, respectively. While these were not statistically significant differences in age, it is possible that age-related functional differences in postural control may have contributed to our observations. Finally, while we attempted to minimize the influence of behavioral factors such as hunger and sleepiness, these factors can easily influence infant behavior.

Future work should explore the validity, predictive ability, and sensitivity to change (over time and with intervention) of early measures of postural control in infants at risk for physical disability. This includes evaluation of postural control measures during specific infant motor behaviors (unilateral and bilateral kicking and reaching) and additional measures to those studied here, such as composite indices and nonlinear as well as linear measures.

## Conclusion

Quantitative measures of spontaneous infant movement may have promise to distinguish early in life between infants who are at risk for motor impairment or physical disability and those who will demonstrate typical motor control. With precise technology, infant supine postural control is an easy-to-collect quantitative functional measure and should be considered in future work exploring the concurrent validity and predictive value of objective measures of infant movement. The hypothesis that COP path length may be a potential early marker of postural instability and motor control impairment needs further confirmation and further investigation to elucidate the responsible neuromotor mechanisms.
